# New factors in mammalian DNA repair—the chromatin connection

**DOI:** 10.1038/onc.2017.60

**Published:** 2017-04-10

**Authors:** G Raschellà, G Melino, M Malewicz

**Affiliations:** 1 ENEA Research Center Casaccia, Laboratory of Biosafety and Risk Assessment, Rome, Italy; 2 Department of Experimental Medicine & Surgery, University of Rome Tor Vergata, Rome, Italy; 3 MRC Toxicology Unit, Hodgkin Building, Leicester, UK

## Abstract

In response to DNA damage mammalian cells activate a complex network of stress response pathways collectively termed DNA damage response (DDR). DDR involves a temporary arrest of the cell cycle to allow for the repair of the damage. DDR also attenuates gene expression by silencing global transcription and translation. Main function of DDR is, however, to prevent the fixation of debilitating changes to DNA by activation of various DNA repair pathways. Proper execution of DDR requires careful coordination between these interdependent cellular responses. Deregulation of some aspects of DDR orchestration is potentially pathological and could lead to various undesired outcomes such as DNA translocations, cellular transformation or acute cell death. It is thus critical to understand the regulation of DDR in cells especially in the light of a strong linkage between the DDR impairment and the occurrence of common human diseases such as cancer. In this review we focus on recent advances in understanding of mammalian DNA repair regulation and a on the function of PAXX/c9orf142 and ZNF281 proteins that recently had been discovered to play a role in that process. We focus on regulation of double-strand DNA break (DSB) repair via the non-homologous end joining pathway, as unrepaired DSBs are the primary cause of pathological cellular states after DNA damage. Interestingly these new factors operate at the level of chromatin, which reinforces a notion of a central role of chromatin structure in the regulation of cellular DDR regulation.

## Introduction

**Figure 1:**
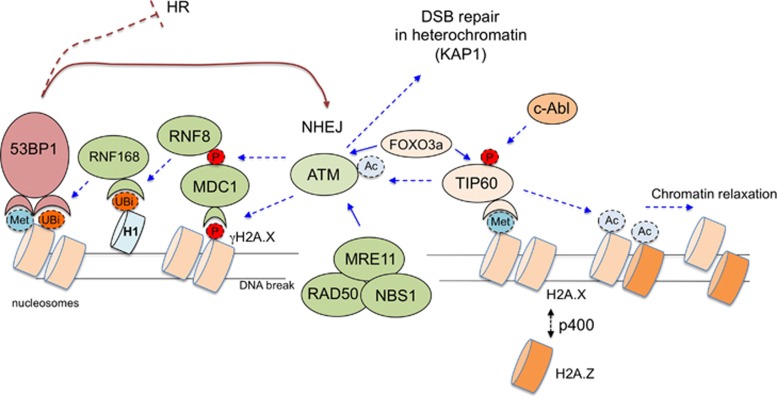
A simplified scheme of chromatin changes in response to double-strand break (DSB) occurrence highlighting a central role of the ATM kinase. DSBs are recognized by the MRN complex (MRE11; RAD50; NBS1), which leads to activation of the ATM kinase.^111^ In parallel DNA damage-induced chromatin changes lead to c-Abl tyrosine kinase activation. C-Abl phosphorylates TIP60 (also known as KAT5) acetyltrasferase, which acetylates ATM to elicit full activation of this kinase.^112^ Interaction between ATM and TIP60 is facilitated by FOXO3A transcription factor.^60^ Binding of TIP60 to methylated histones (predominantly H3K9me3) is required for its action on ATM and other histone substrates (e.g., H4). TIP60 acetylates histones leading to the formation of open relaxed chromatin structure.^18^ This step is facilitated by p400 histone chaperone-mediated variant histone exchange at DSBs (H2A.X is replaced by HA2.Z) occurring in close proximity to DSBs (up to ca 3,5 kb away from a DSB).^39^ The main substrate of ATM kinase is histone H2A.X and phospho-H2A.X (termed γH2A.X) spreads away from the DSBs into megabase sized domains.^113^ Phospho-H2A.X is recognized by MDC1 adapter protein, which is also the substrate of ATM.^114, 115^ Phospho-MDC1 in turn recruits RNF8 ubiquitin ligase.^116^ Linker histone H1 is the main substrate of RNF8 and ubiquitinated H1 is recognized by RNF168 ubiquitin ligase.^117^ RNF168 ubiquitinates histone H2A on K13/15, which facilitates recruitment of the key adapter protein 53BP1.^118^ Stable binding of 53BP1 also requires its association with methylated histone H4 (K20me2).^119, 120^ Of note histone methylation is a constitutive chromatin mark, whereas both histone acetylation and ubiquitination are dynamic DNA damage-induced modifications.^121^ 53BP1 serves as critical regulation of DSB repair pathway choice and promotes NHEJ repair by inhibiting DNA resection (a critical step in homologous recombination (HR) repair).^122^ ATM is also implicated in the repair of heterochromatin DSBs by phosphorylating KAP1 protein, which in turn promotes opening of heterochromatin to allow for the access of the DNA repair machinery.^123^

Cellular DNA is undergoing a constant damage due to the action of endogenous free radicals, replication errors and exogenous sources of radiation.^1^ Cells have evolved a sophisticated set of DNA damage sensors that serve to detect DNA damage and launch activation of several stress response pathways collectively referred to as the DNA damage response (DDR).^2^ To prevent deleterious consequences of replication through damaged DNA, cells typically temporarily arrest cell cycle (checkpoint activation),^3, 4, 5^ inhibit transcription and translation and initiate DNA repair. At the face of very extensive DNA damage cells can also active a program of self-destruction termed apoptosis.^6, 7^ Persistent DNA damage on the other hand can lead to cellular senescence.^7^ The ultimate cell fate following DNA damage will be determined by the extent, type and severity of DNA damage experienced by the cell. The efficiency of DNA repair plays a paramount importance in cellular response to DNA damage induced by drugs that are frequently used in cancer chemotherapy.^7, 8, 9, 10, 11, 12, 13^ The repair of DNA requires lesion recognition by specialized sensor molecules but also importantly adjustment of chromatin structure in the lesion vicinity.^14^ This DNA damage-induced chromatin changes serve several purposes. Marking of chromatin at the lesion creates a recognition signal for the cellular machinery to assemble at the site to initiate the repair process. Secondly chromatin changes at the DNA facilitate the repair process by allowing access of various repair and signaling complexes. Thus chromatin-level responses lie at the heart of cellular coordination of the DDR.^15^ Chromatin changes in response to DNA damage occur at several distinct levels such as: nucleosome remodeling, variant histone exchange, non-histone chromatin protein mobility alteration and histone tail post-translational modification.^16^ In this review we will summarize several recent findings of new protein factors and mechanisms governing the regulation of cellular DNA repair with emphasis on double-strand DNA break (DSB) repair and highlighting the central role of chromatin-level regulation (Figure 1).

## New findings in double-strand DNA break repair regulation—focus on non-homologous end joining

**Figure 2:**
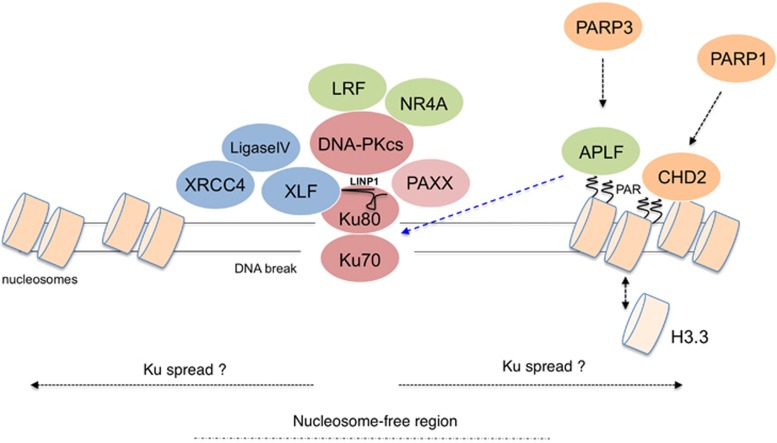
A simplified model for DSB repair mechanism via non-homologous end joining (c-NHEJ) in chromatin with emphasis on the role of poly-ADP-ribosylation (PAR). Double-strand DNA break (DSB) is initially bound by Ku dimer. DNA-bound Ku undergoes an allosteric change to recruit other effectors of the c-NHEJ DSB repair pathway: DNA-PKcs, PAXX, XLF and XRCC4/Ligase IV. Ku can potentially spread away from the DSB. The interaction between Ku and DNA-PKcs is further stabilized by various factors such as LRF and LIMP (ncRNA). This figure includes some of the known chromatin-remodeling steps that occur at DSBs such as generation of limited nucleosome-free region and PAR-dependent variant histone exchange (see Figure 1 for a more extensive description of DSB-related chromatin changes). PAR-dependent H3.3 variant histone exchange is promoted by PARP-1 in conjunction with the CHD2 chromatin remodeller and is required for stable association of Ku with DSBs. Additional accessory c-NHEJ factor is the APLF protein that binds to PARsylated chromatin (in this case PARsylation is catalysed by PARP3) and interacts with Ku to enhance the stability of repair complexes on chromatin. NR4A nuclear orphan receptors bind DNA-PKcs and are able to promote DSB repair.

Although DNA can be damaged in several distinct ways the formation of DSBs is considered the most dangerous to the cell.^17^ This is due to complexity of the DSB repair process and a potentially dramatic inhibition of replication and transcription the DBSs impose. There are two general strategies mammalian cells utilize to deal with double-strand breaks in DNA: non-homologous end joining (NHEJ) and homology-directed repair.^18, 19^ Here we focus on the recent progress in understanding NHEJ mechanism and direct those interested in learning the latest on homology-directed repair regulation to excellent recent reviews published elsewhere.^20^ NHEJ repair process is aimed at restoration of the linear DNA structure across the break with the accuracy of the repair playing a less critical role.^21, 22^ A specialized DSB sensor Ku70/Ku80 heterodimer, which has a high affinity for double-stranded DNA, is recruited to DSBs within seconds to initiate the classical NHEJ repair process.^3^ Binding to DNA induces an allosteric change within the Ku complex, which results in recruitment of the DNA-PKcs protein. DNA-PKcs has a protein kinase activity and is activated by the presence of free DNA ends. Collectively Ku/DNA-PKcs complex is called the DNA-PK kinase and its activity is triggered by DSBs. DNA-PK bound to DSBs orchestrates the DNA repair process by allowing DNA end modifying enzymes to access to DSBs to generate ligatable DNA ends (Figure 2). Additional core components of NHEJ are XLF/Cernunnos and XRCC4/Ligase IV complex, which are recruited to DNA in Ku-dependent fashion. Independently of Ku interaction, XLF and XRCC4 also are able to form long filamentous assemblies at DSBs, which contribute to proper DSB alignment, whereas Ligase IV is responsible for the final ligation reaction.^23, 24^

**Figure 3:**
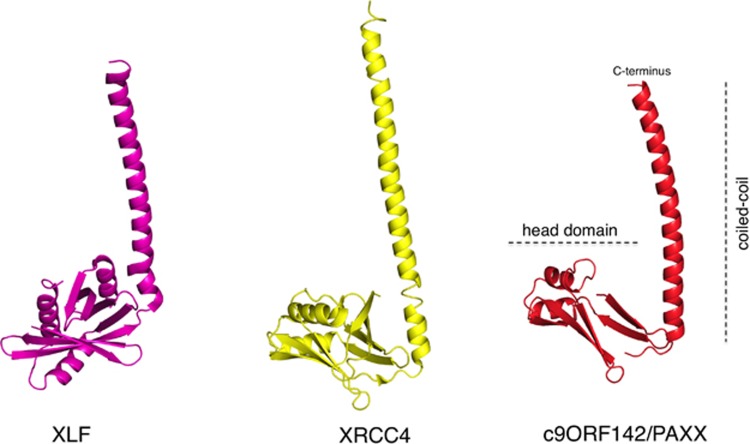
Newly discovered c-NHEJ factor c9orf142/PAXX structurally resembles XRCC4. Figure shows computer models of XLF, XRCC4 and c9orf142/PAXX (adapted from Craxton *et al.*, 2015). These proteins typically form dimers, however, here for simplicity and to highlight their structural similarity monomers are depicted. For XLF C-terminal portion of the coiled-coil has been shortened. The overall structure of a typical XRCC4 paralog includes a globular head domain, a centrally located coiled-coil and the C-terminal region (CTR, not shown). The CTRs are intrinsically disordered and not visible on available crystal structures. The head domain of XLF and XRCC4 is responsible for the formation of XLF/XRCC4 filaments, whereas the head domain of PAXX has an unknown function. The coiled-coils include regions necessary for dimerization. CTRs of XLF and PAXX mediate the interaction with Ku (not shown).

XLF/Cerunnos was identified in the year 2006^25, 26^ and was considered to be the final core component of NHEJ to be discovered. It was thus remarkable that in 2015 three independent papers reported a discovery of a new NHEJ factor—c9orf142—subsequently named PAXX (paralogue of XRCC4 and XLF)^27, 28^ or XRCC4-like small protein.^29^ It remains unclear as to why PAXX factor had not been found earlier, however, a possibility exist that it plays a minor role in rodent cell lines, which were the foundation for the early NHEJ characterization efforts.^30^ Clearly humans evolved a more prominent role for some NHEJ proteins highlighted by essential functions for Ku and DNA-PKcs in human cells.^31^ The discovery of PAXX uncovered an unforeseen complexity in NHEJ activity regulation. Although PAXX tertiary (but not primary) structure resembles that of both XLF and XRCC4 and thus it is considered their paralog (Figure 3), PAXX actually associates with DNA–PK complex (through specific contacts with Ku subunit; Figure 2). Furthermore evolutionary presence of PAXX is limited and, unlike XLF and XRCC4, it is not found in fungi, worms and the fruit flies. The fact that PAXX primary structure is most conserved in vertebrates (some simpler organism bear a more diverged homolog of PAXX^27, 28, 29^) suggested an important role for this protein in somatic DNA recombination. Two recent papers confirmed this prediction by showing that in pro-B cells PAXX is essential for variable, diversity, joining recombination, although surprisingly its function is normally masked by the dominant activity of XLF.^32, 33^ Accordingly unlike the single knockouts PAXX/XLF double knockout B cells have a dramatic defect in variable, diversity, joining recombination. Furthermore generation of PAXX/XLF double knockout mice have been recently reported.^34^ Similarly to both XRCC4 and Ligase IV, single-knockout mice PAXX/XLF-combined mutant animals were not viable demonstrating important yet overlapping roles for PAXX and XLF in mammalian NHEJ. These findings explain a long-standing puzzle of a relatively weak phenotype associated with XLF loss.^35^ Thus it appears that PAXX and XLF have partially overlapping and redundant function in mammalian DSB repair. Based on *in vitro* studies and human cellular models PAXX functions by stimulating the activity of Ligase IV in promoting blunt-end DNA ligation and in conjunction with XLF stimulates the non-compatible (gapped) DNA end ligation.^28^ Both activities are strictly dependent on direct contacts between the conserved PAXX C-terminus and the Ku DNA sensor. PAXX interacts with DNA-bound Ku, however, the presence of DNA also triggers interaction of Ku with all other NHEJ components.^29^ It was therefore not possible up to this point to characterize the domain of Ku with which PAXX makes a direct contact. Another mystery of PAXX protein is the function of its head domain. Thus, a lot more work is needed to fully understand the extent of PAXX contribution to NHEJ. Molecular modeling predicted PAXX to potentially form filaments analogous to XLF/XRCC4 structures and furthermore PAXX is essential for maintaining appropriate levels of all other NHEJ factors on chromatin after DNA damage.^27^ It remains to be determined of whether these PAXX functions are related. Nevertheless the action of PAXX at the level of chromatin is an interesting observation that highlights the growing importance of chromatin-level regulation for NHEJ.

## The importance of chromatin for NHEJ regulation

Early biochemical data obtained *in vitro* with pure proteins and naked DNA suggested a simple 1:1 Ku/DNA end binding stoichiometry.^36^ This model had recently been validated *in vivo* by a discovery of small Ku foci assembling on DSBs in irradiated cells.^37^ However, other reports employing chromatin immunoprecipitation observed a spread of Ku (and of DNA-PKcs^38^) up to 5 kb away from the break on chromatin flanking DSBs.^39^ The discrepancy likely lies in the methodology, as the visualization of small Ku foci is only possible once the majority of chromatin-bound Ku is pre-extracted prior to fixation, which leaves a possibility that some RNAse-sensitive fraction of Ku is able to spread away from the DSB. DNA-damage-induced Ku chromatin loading can also be observed with cellular fractionation techniques^27^ and via laser based microirradiation.^40^ However, neither of these assays provides DSB distance information in relation to Ku chromatin binding nor are such assays informative of the protein stoichiometry involved. It thus currently unclear of whether Ku can spread on chromatin at DSBs. However, in contrast to simple Ku/DSB interaction model derived from *in vitro* studies the loading and maintenance of Ku on chromatin in living cells appears to be regulated. What are the mechanisms determining the extent of Ku loading on chromatin? While at present not entirely clear several observations point to a possible role of poly-ADP-ribosylation in this process. Accordingly, cellular DNA breaks are also detected by several poly-ADP-ribosylases^41, 42^ (PARPs; most notably PARP1, 2 and 3)^43^ and binding of these enzymes to DNA strand breaks triggers their activation with subsequent catalysis of long poly-ADP-ribose (PAR) chains at the lesion. PAR is a NAD-derived branched polymer composed of ADP-ribose units and therefore PAR has a certain chemical resemblance to both RNA and DNA. PAR can be covalently attached to various proteins at DNA lesions with histones being the most prominent target,^44^ however, the exact amino acid positions of this modification on histone proteins had not been precisely mapped. PAR polymer is specifically recognized by specialized protein domains present in various DNA repair factors such as the APLF protein,^45, 46^ which performs an accessory/non-essential role in mammalian NHEJ reactions. In the context of DSB repair APLF had been shown to contribute the NHEJ execution at the chromatin level.^42^ APLF utilizes a PAR-binding zinc-finger (ZF) domain to bind PAR and a centrally located MID domain to bind Ku.^47^ By coupling these abilities APLF acts as a scaffolding molecule able to enhance the stability of NHEJ repair complexes in the context of PARsylated chromatin. A strong support for a major PAR contribution to NHEJ comes from studies in the slime mold *Dictyostelium discoideum.*^48^ This model organism apparently lacks an APLF homolog, however, its Ku70 homolog bears a PAR-binding ZF domain, which is essential for Ku functionality in *Dd* NHEJ. Of note, in *Dictyostelium* the APLF function may be partly taken over by other proteins that possess additional domains of significant homology to human APLF, namely XRCC1^49^ and APL.^50^ Despite these findings in mammalian DSB repair models the contribution of PARP1 to classical NHEJ had been a subject to an intense debate.^43^ The main argument against the role of PARP1 in NHEJ was a relatively minor phenotype of PARP1 mutant mice in terms of radiation sensitivity and DSB repair impairment and inconsistent phenotypes associated with PARP-1 deficiency observed in cellular settings.^51^ Furthermore, a strong case emerged for a participation of PARP1 in an alternative form of NHEJ,^43^ which operates under circumstances of defective or suppressed classical NHEJ. Notably the latest findings suggest a possible mechanism of PARP1 contribution to classical NHEJ.^52^ Accordingly this model proposes a PARP1/CHD2/H3.3-dependent chromatin-remodeling step being necessary for proper Ku and XRCC4 loading on chromatin at DSBs. Such a model is consistent with data obtained using DSB inducing enzymes, where a certain level of nucleosome disruption at DSBs had been found necessary for XRCC4 loading.^53^ The definition of PARP-1 function in c-NHEJ explains several independent observations linking PAR, PARP1 and Ku in classical NHEJ regulation in human cells (Figure 2). There is evidence for other PAR-dependent mechanisms operating in mammalian DSB repair,^54, 55^ however, at present the exact relationship of these factors to known DNA repair pathways awaits further clarification. Finally Ku/chromatin interaction is subject to regulation via chromatin acetylation and specific nucleosome remodeling at DSBs.^39, 40, 56^ In summary although the exact Ku stoichiometry at DSBs and the neighboring chromatin is still uncertain the binding of Ku to DNA lesions in chromatin can be regulated by a variety of factors.

Similarly to Ku chromatin recruitment Ku/DNA-PKcs complex formation at DSBs also appears regulated at various levels. For example an lncRNA molecule (LINP1) had recently been described that binds to Ku80 and facilitates its interaction with DNA-PKcs.^57^ Of note as mentioned above the method for detection of Ku foci involves a RNAse treatment step,^37^ which raises a question of whether the bulk of chromatin-bound Ku is retained at this structure via specific RNA-Ku interactions. Other examples of regulation of this step of the repair complex assembly include a case of sequence-specific transcription factor (TF) LRF.^58^ It has been demonstrated that LRF directly binds DNA-PKcs protein and somehow increases its association with Ku contributing to maintenance of proper levels and stoichiometry of DNA-PK holoenzyme on chromatin. Strikingly in the genetic absence of LRF, the overall kinase activity of DNA-PK is diminished, which is a dramatic finding given a large excess of Ku and DNA-PKcs levels in cells. A potential explanation for this is that the rodent cell culture system (mouse embryonic fibroblasts) used in that study is characterized by lower steady-state DNA-PK levels than an average human cell.^59^ Under these specific circumstances efficient DNA-PK activation *in vivo* might require additional accessory factors, which could be a subject to additional signal- or tissue-specific control. Interestingly, a similar regulatory model of DNA-damage kinase activation with participation of bridging factors in a form of sequence-specific transcriptional regulators have recently also been described for ataxia telangiectasia-mutated (ATM) kinase.^60^ ATM is a very prominent DDR-activated kinase that functions primarily in signal transduction and DSB repair.^61^ ATM activity is vital for several aspects of DDR regulation.^61^ Most notable is its ability to promote various DSB repair pathways such as homologous recombination (through phosphorylation of a key resection factor CtIP^62^), the repair of DSBs in heterochromatin (by phosphorylation of KAP1 protein^17^) and c-NHEJ (in that process ATM is redundant with XLF^63^). ATM is also very prominent in regulating DSB-related signaling (including checkpoint induction), chromatin remodeling and histone tail post-translational modifications (Figure 1). Recently FOXO3A TF had been found to promote association of ATM with TIP60 acetyltransferase (Figure 1), which is one of the many avenues for optimal ATM activation after DNA damage.^60^ Interestingly NR4A nuclear orphan receptors can also bind and collaborate with DNA-PKcs in DSB repair thus expanding the list examples of TFs involved directly in DNA repair.^64^ Collectively these observations suggest that a larger collection of regulatory factors exist in living cells to control the activity and assembly of protein complexes containing DNA damage-activated protein kinases. Interestingly the above mentioned mechanisms seem most relevant *in vivo* in the native chromatin context (summarized in Figures 1 and 2), which highlights the need for more extensive efforts to study the DNA repair regulation under physiological conditions in chromatin whenever possible.

## Contribution of transcription factors to DDR regulation.

Importantly, however, several prominent DDR factors have a capacity to regulate transcription and contribute to genomic stability and other processes^65^ indirectly via that mechanism. Examples are numerous and include such key DDR proteins such as 53BP1,^66^ BRCA1,^67^ CtIP,^68^ KAP1,^69^ PTIP,^70, 71^ DNA-PKcs,^72^ ATM,^73^ ATMIN,^74^ CHD4^75^ and many others. Reciprocally several TFs that evolved to control gene expression have been increasingly associated with direct regulation of DDR related processes at DNA lesions^76^ (see above for LRF^58^). With regard to the DDR regulation a given protein factor can operate both at the lesion and through indirect means by affecting transcription of direct effectors.^76, 77^ Interestingly for many of such multifunctional factors their direct function in DDR and in transcription can be separated genetically and involve distinct domains and interaction partners (53BP1 being an excellent example^66^). Interestingly a recent study of TF recruitment to DNA damage sites further blurred the division between a transcriptional regulation and a direct action at DNA lesions by showing that some TFs use their DNA-binding domain for interaction with DNA exposed at the damaged break.^78^

TFs play a central role in controlling virtually all biological processes occurring in the cells including cell cycle progression,^79^ maintenance of intracellular metabolic and physiological balance, cellular differentiation, developmental time courses^80, 81, 82^ and DNA repair.^76^ Surprisingly, the exact number of human TFs remains still undefined. This lack of knowledge depends at least in part, on the definition of TF that varies in different studies attempting to catalog this class of genes.^83, 84^ For instance, transcription co-factors (that is, proteins which modulate the activity of other transcription controllers but are unable to act independently), are not always registered in the list of 'real' TFs. Vaquerizas *et al.* utilized the definition of TFs as a class of proteins that binds DNA in a sequencespecific manner, but are not enzymatic or do not form part of the core initiation complex.^85^ According to this classification, the Authors listed a high confidence dataset of approximately 1400 genomic loci that encode TFs. This number is expected to increase to an upper bound of 1700–1900 TF-coding genes in the human genome.

A distinctive part in the architecture of TFs is the DNA-binding domain that has been utilized for their classification. The most represented DNA-binding domain among human TFs is the two-cysteine two-histidine ZF followed by homeodomain and helix-loop-helix.^86^ Some types of DNA-binding domains are frequently used in the cell for the execution of specific functions. For example, the homeobox domains are essential for morphogenesis, organogenesis and establishment of body plan in vertebrates.^87, 88^ Notwithstanding the evolutionary conserved nature of most TF families in vertebrates, the two-cysteine two-histidine ZF family stands out as a prominent exception. In fact, many ZF factors derive from duplication throughout the evolution.^89^ Consequently, this evolutionary dynamics contributes to their difficult classification in functionally distinct subfamilies. Typically, ZF factors possess an array of two-cysteine two-histidine motifs that defines a polydactyl structure in which each finger binds three adjacent nucleotides at the DNA recognition sites. The polydactyl structure further complicates the ZF factors binding specificity since adjacent motifs influence each other’s DNA binding.^89^ Recently, it has been demonstrated that the ZF factor Zfp335 possesses two major DNA-binding domains comprising distinct ZF clusters.^90^ Intriguingly, each domain encodes a different sequence specificity implying that binding to multiple sequence motifs could be relevant for specific gene regulation. Usage of multiple motifs may result in a more efficient targeting of TFs to their binding sites and could be important for context-dependent function. Thus, ZF factors are expected to have a broad number of targets whose functional significance is strictly dependent on their binding topography, which in turn can vary depending on the physiological state of the cell. Indeed, ZF factors that were initially defined as specific for regulation of one pathway, to a deeper analysis, revealed a more pleiotropic function. This is the case of CTCF, an 11 ZF protein initially described as a negative regulator of Myc expression.^91^ Further analysis highlighted a much broader ability of CTCF in recognizing different targets through the combinatorial use of its 11 ZFs. *In vivo* mapping revealed that CTCF reads sequence diversity through ZF clustering. In fact, ZFs 4–7 anchor CTCF to most of the targets containing the core motif while ZFs 1–2 and ZFs 8–11 clusters recognize non-conserved flanking sequences.^92^ As a consequence of this combinatorial modality of recognition, the targets of a single-ZF factor often refer to a variety of cellular pathways, which are not obviously interrelated.

The plasticity of TFs in recognizing their targets highlighted another somehow unexpected ability of this broad family of genes. As mentioned earlier, a recent study that utilized epitope-tagged proteins for localization to sites of DNA damaged by UV laser microirradiation, found >120 proteins that localize to damaged chromatin. Intriguingly, ~70% of the TFs included in this study, were able to migrate to damaged DNA.^78^ Of interest, in a set of 35 TFs evaluated for their presence at the DNA-damaged sites, 13 were ZF factors. The integrity of the DNA-binding domain seems to be a necessary condition for recognition of damaged DNA by TFs. Relocation of the TFs at the DNA-damaged sites relies on chromatin decompaction, which in turn is dependent on PARP1 activity. The mechanism(s) through which PARP1 decompacts chromatin until recently were completely unclear. One example recently emerged in the context of NHEJ regulation (see above^52^). Another possible mechanism could be that PARP1 somehow recruits other chromatin remodelers, however, silencing of remodelers such ALC1, CHD4, INO80, TIP60, KAT2A, SMARCC1, SMARCC2, BAZ1B, EZH2, SUZ12 and p300 did not affect recruitment of TFs on damaged DNA. This result does not rule out the existence of a redundant mechanism, which implies the synergistic action of a combination of factors. Alternatively, PARP1-dependent chromatin remodeling could be dependent on a yet unidentified factor. Since some TFs are endowed with the ability of recruiting chromatin remodelers,^93^ a direct contribution of TFs to this task can also be hypothesized at the DNA-damaged sites.

## ZNF281 a multi-task zinc finger protein

**Figure 4:**
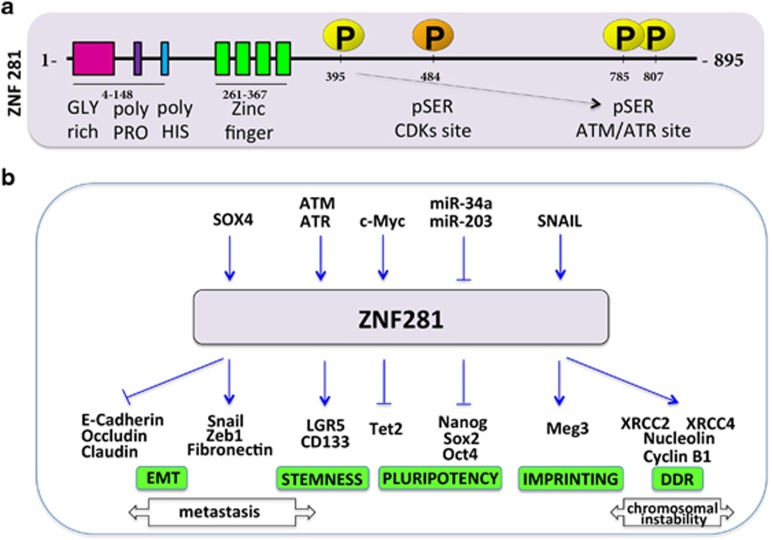
Summary of the structure and functions of the ZNF281 protein. (**a**) Schematic presentation of the human ZNF281 protein. Boxes represent distinct functional domains of ZNF281. ZNF281 is a substrate of ATM/ATR DNA damage-activated kinases (yellow P circles) as well as CDKs (Cyclin-dependent kinases; orange P circle), however, the impact of ATM/ATR phosphorylation or CDK phosphorylation on ZNF281 function is unknown. (**b**) expression of ZNF281 is regulated by a variety of protein factors and miRNAs (shown in the upper part of panel b). ZNF281 (zfp281) can regulate positively or negatively a number of target genes (blue arrows in the lower part of panel b). ZNF281 and its murine homolog zfp281 are involved in the induction of epithelial–mesechymal transition (EMT) of colon cancer cells, in control of cellular stemness, in the imprinted expression of the Meg3 polycistron and in regulation of genes involved in the DNA-damage response (DDR). Transcription repression of Nanog and Tet2 is achieved through recruitment of the histone deacetylase HDAC2. Arrow-headed and bar-headed lines indicate activation and inhibition respectively.

The molecular structure of ZNF281 (aliases: ZBP-99, ZNP-99) is characterized by 4 two-cysteine two-histidine ZFs located near the N-terminal of the protein which has a mass of 99 kD^94^ (Figure 4). The prominent functional feature of ZNF281 resides in its ability to bind to GC-rich sequences located in the regulatory regions of many genes where its binding often results in transcriptional promotion or repression of the targets.^95, 96^ After approximately a decade during which its function(s) in the cell remained undetermined, zfp281 (the mouse homolog of ZNF281) was recognized as a component of a protein interaction network for pluripotency of embryonic stem cells.^97^ Further analysis of its role demonstrated that zfp281 is a transcriptional repressor of Nanog, an essential gene for cellular stemness.^98^ Indeed, genetically ablated Zfp281 null ESCs unambiguously argue for a repressor function of Zfp281 in regulation of major stem cell factors among which Sox2 and Oct4.^98^ Of interest, zfp281 exerts its inhibitory activity by recruiting the NuRD repressor complex on Nanog promoter.^93^ The latter mechanism was also demonstrated in the transcriptional repression of the TET2 gene.^99^ In this context, zfp281 recruits HDAC2 (a component of the NuRD complex) causing histone H3 deacetylation and chromatin compaction. In addition, zfp281 also promotes the transcription of miR302/267, which in turn post-transcriptionally represses TET2 expression. The overall result of TET2 repression is the maintenance of a state of primed pluripotency further demonstrating the importance of zfp281 in regulating cellular stemness. In mouse embryonic stem cells, zfp281 recruits AFF3 to the Meg3 enhancer within the imprinted Dlk1-Dio3 locus, to regulate the allele-specific expression of the Meg3 polycistron.^100^ In human colon carcinoma cells, ZNF281 controls epithelial–mesenchymal transition (EMT).^95^ Indeed, Snail, an EMT-associated protein, promotes the expression of ZNF281 and represses miR-34a/b/c, a target of ZNF281. The latter, in turn, activates the transcription of Snail thus establishing a feed-forward regulatory loop. Indeed, ZNF281 itself is able to induce EMT by controlling the expression of several EMT-associated genes and by down-regulating epithelial markers such as Occludin, Claudin and E-Cadherin. Intriguingly, the same study demonstrated that c-Myc-induced EMT is dependent on ZNF281 expression. In line with its involvement in stemness control, ZNF281 also induces the stemness markers LGR5 and CD133, and increases sphere formation.^95^ These results highlight a dual role of ZNF281 in controlling cellular stemness and in induction of EMT in tumors (and possibly also in normal cells) thereby suggesting the plasticity of this TF, whose function can differ depending on the physiological (or pathological) state of the cell (Figure 4b).

Transcriptional regulation of ZNF281 remains largely unknown. Beside the already mentioned Snail,^95^ SOX4, which is expressed in many human malignancies, promotes the transcription of ZNF281.^101^ Chromatin immunoprecipitation followed by sequencing analysis demonstrated that SOX4 binds to DNA sequences in the proximity of ZNF281 gene, suggesting a binding-dependent regulation. The expression of ZNF281 is post-transcriptionally repressed by miR34a through a p53-dependent mechanism^95^ and by miR203.^102^ Regulation of ZNF281 could occur also through epigenetic mechanisms since differential methylation of ZNF281 gene has been recently described (Petrus *et al.* 2016).^103^ Of interest, the ZNF281 protein is phosphorylated by ATM and ATM- and ataxia telangiectasia Rad3-related (ATR) kinases after DNA damage^104^ (Figure 4a). In humans, the closest ZNF281 homolog is ZBP-89, which is frequently over-expressed in human cancer cells, where it can efficiently induce apoptosis through p53-dependent and -independent mechanisms.^105^ A human ZBP-89 splice isoform ZBP-89-DN, which lacks amino terminal residues 1–127 of the full length protein, has also been identified, which predisposes the colon to colitis.^106^

## ZNF281 in the DNA damage response

We recently reported an increase of ZNF281 expression after genotoxic stress by DNA damage inducing drugs.^107^ We observed this phenomenon in p53-proficient and -deficient tumor cells, as well as in normal primary keratinocytes and in mouse skin *in vivo*. The increase of ZNF281 following DNA damage occurs through dominant, p53-independent mechanism, which does not rely on transcriptional regulation. A significant delay in DNA repair in cells silenced for ZNF281 expression suggests that the expression of ZNF281 could have functional implications in DNA repair. Among several DDR-associated genes whose expression is affected by ZNF281, XRCC4 and XRCC2 two components of the NHEJ and homologous recombination DNA repair pathways respectively,^108^ are promoted by ZNF281 through a mechanism dependent on its binding to their promoters. Of interest, Nucleolin, a protein that mediates nucleosome disruption critical for DNA double-strand break repair^109^ and which is a known target of c-Myc,^110^ is not transcriptionally activated by ZNF281, which acts instead as a co-factor of c-Myc in Nucleolin activation. Together these results demonstrate for the first time, that ZNF281 is involved in the DDR by its classical function of TF that controls the expression of other genes (Figure 4b). Nevertheless, ZNF281 could give its contribution to DNA repair through a less obvious mechanism. As mentioned before, among the TFs that were demonstrated to move to the sites of damaged DNA there is ZNF281.^78^ What could be the function of ZNF281 on the sites of damaged DNA? ZNF281 could recruit chromatin remodelers to the sites of broken DNA by a mechanism similar to that utilized on the promoters of Nanog^93^ and TET2^99^ (Figure 4b). We can also speculate that ZNF281 interacts with core components of the DNA repair machinery and contribute to the role of the recently characterized XLF and PAXX in maintaining appropriate levels of all other repair factors on chromatin. Although our knowledge on the involvement of ZNF281 in NHEJ as well as other repair pathways is still limited, future work could disclose other relevant and unexpected roles for this factor in DNA repair.

## Conclusions and future directions

We conclude that a number of recently discovered factors in mammalian DNA repair operate at the level of chromatin. The recent addition of proteins such PAXX and possibly ZNF281 to the overall framework of the repair mechanisms, underscores our still incomplete knowledge of the molecular details underlying the chromatin modifications which occur during the DDR. As many clinically relevant cancer treatments rely on DNA damage induction in tumor tissue a better understanding of contribution of chromatin changes to DDRs will be instrumental in further development of such therapies.

## Abbreviations

C2H2: two cysteine two histidine
*Dd*: *Dictyostelium discoideum*
DDR: DNA-damage response
DNA-PK: DNA-dependent protein kinase
DSB: double-strand breaks
EMT: epithelial–mesenchymal transition
lncRNA: long non-coding RNA
NHEJ: non-homologous end joining
PAR: poly-ADP-ribose
TFs: transcription factors
ZFs: zinc fingers.
